# Postural alignment differences in children with urinary incontinence

**DOI:** 10.1038/s41598-026-65216-x

**Published:** 2026-08-03

**Authors:** Damla Korkmaz Dayican, Busra Palaz, Ozge Celiker Tosun, Arzu Razak Ozdincler, Ali Tekin, Sibel Tiryaki, Ibrahim Ulman

**Affiliations:** 1 Department of Physiotherapy and Rehabilitation, Faculty of Health Sciences, İzmir Tınaztepe University, İzmir, Turkey; 2 Department of Physiotherapy and Rehabilitation, Faculty of Health Sciences, İzmir Bakırçay University, İzmir, Turkey; 3 Department of Physiotherapy and Rehabilitation, Faculty of Physical Therapy and Rehabilitation, Dokuz Eylül University, İzmir, Turkey; 4 Department of Physiotherapy and Rehabilitation, Faculty of Health Sciences, Fenerbahçe University, İstanbul, Turkey; 5 Division of Pediatric Surgery, Department of Surgical Medical Sciences, Faculty of Medicine, Ege University, İzmir, Turkey

**Keywords:** Urinary incontinence, Enuresis, Daytime incontinence, Postural alignment, Core muscles, Diseases, Health care, Medical research, Signs and symptoms, Urology

## Abstract

This study examined whether postural alignment differs between children with urinary incontinence and asymptomatic children. Participants were children aged 5–14 with daytime urinary incontinence (*n* = 14), monosymptomatic nocturnal enuresis (*n* = 13), non-monosymptomatic nocturnal enuresis (*n* = 13), and asymptomatic children (*n* = 13). Postural alignment was assessed with the PostureScreen Mobile application. Lower urinary tract symptoms were evaluated using the Dysfunctional Voiding and Incontinence Scoring System, Vancouver Bladder/Bowel Dysfunction Questionnaire, and Childhood Bladder and Bowel Dysfunction Questionnaire. Core muscle function was measured with surface electromyography and ultrasound. Left lateral total body angulation was significantly greater in urinary incontinence subgroups than in the asymptomatic group (*p* = 0.002). Left lateral, right lateral, and posterior hip-pelvis angulations were also higher (*p* = 0.001, *p* = 0.045, and *p* = 0.013), especially in the non-monosymptomatic nocturnal enuresis subgroup. Children with urinary incontinence and greater postural deviation had higher symptom scores. In exploratory analyses, postural alignment parameters correlated with core muscle function in urinary incontinence subgroups (*p* < 0.05). However, these relationships did not remain significant after correction for multiple comparisons. This study showed that children with urinary incontinence had different postural alignment compared to asymptomatic children. The most pronounced differences were found in the hip-pelvis region. Furthermore, a tendency towards left deviation in postural alignment was observed. These changes in postural alignment appeared to be primarily related to the function of core muscles at a peripheral level. However, these exploratory correlations did not remain significant after correction for multiple comparisons. Additionally, the left deviation suggests a possible contribution from lateralisation of postural control at the central level. Therefore, future neuroimaging studies are recommended to investigate the possible relationship between postural alignment changes and the central nervous system.

## Introduction

Urinary incontinence is a common symptom of the lower urinary tract in childhood. The International Children’s Continence Society (ICCS) defines it as involuntary urine leakage in children aged five or older, either persistent or intermittent^1^. Persistent incontinence is typically due to anatomical or neurological causes, while intermittent incontinence may appear as daytime urinary incontinence (DUI) or nocturnal enuresis (NE). Nocturnal enuresis (NE) without daytime symptoms is classified as monosymptomatic (MNE), while NE with daytime incontinence, urgency, or voiding difficulties is classified as non-monosymptomatic (NMNE)^1^.

Central bladder control depends on interactions between several brain regions, and dysfunction in these areas may contribute to lower urinary tract problems^2^. The pontine micturition center, the periaqueductal gray matter, the anterior cingulate gyrus, and the prefrontal cortex work together to maintain continence^3^. Studies indicate that urinary incontinence may involve impaired right prefrontal cortex function^4^. The literature also reports that children with urinary incontinence often display abnormal motor skills, difficulties in speech, coordination, attention, and sleep, as well as academic underachievement, neuromotor delays, minor neurological dysfunction, and short-term memory problems, suggesting impaired prefrontal cortex function^5–7^. Although mainly associated with higher cognitive functions, the prefrontal cortex also plays an important role in regulating posture and balance. Posture is not regulated solely by the spinal cord or cerebellum. The prefrontal cortex also contributes through cognitive control^8^. The prefrontal cortex inhibits inappropriate movements and enables conscious postural adjustments by signaling the motor cortex. Therefore, while it is not the direct ‘architect’ of posture, it is its regulator. Postural disorders may also occur when the prefrontal cortex is disturbed^8,9^.

It has been reported that children with NE exhibit greater lumbar lordosis and pelvic anteversion angles than asymptomatic children^10,11^. It has also been demonstrated that these children have reduced endurance, stability, and mobility in their lumbopelvic muscles^12^. Impairment of the prefrontal cortex in children with urinary incontinence is expected to also affect postural control. However, changes in total body postural alignment in children with incontinence have not yet been adequately investigated. Meanwhile, existing studies have largely focused on incontinence type alone. It remains unclear whether central nervous system involvement, especially of the prefrontal cortex, influences the peripheral muscular system. Therefore, the primary aim of the present study was to determine whether postural alignment differs between children with urinary incontinence and asymptomatic children. The secondary aims were to examine the effect of incontinence type on postural alignment and to assess the relationship between postural alignment, symptoms, and core muscle function.

## Results

**Fig. 1:**
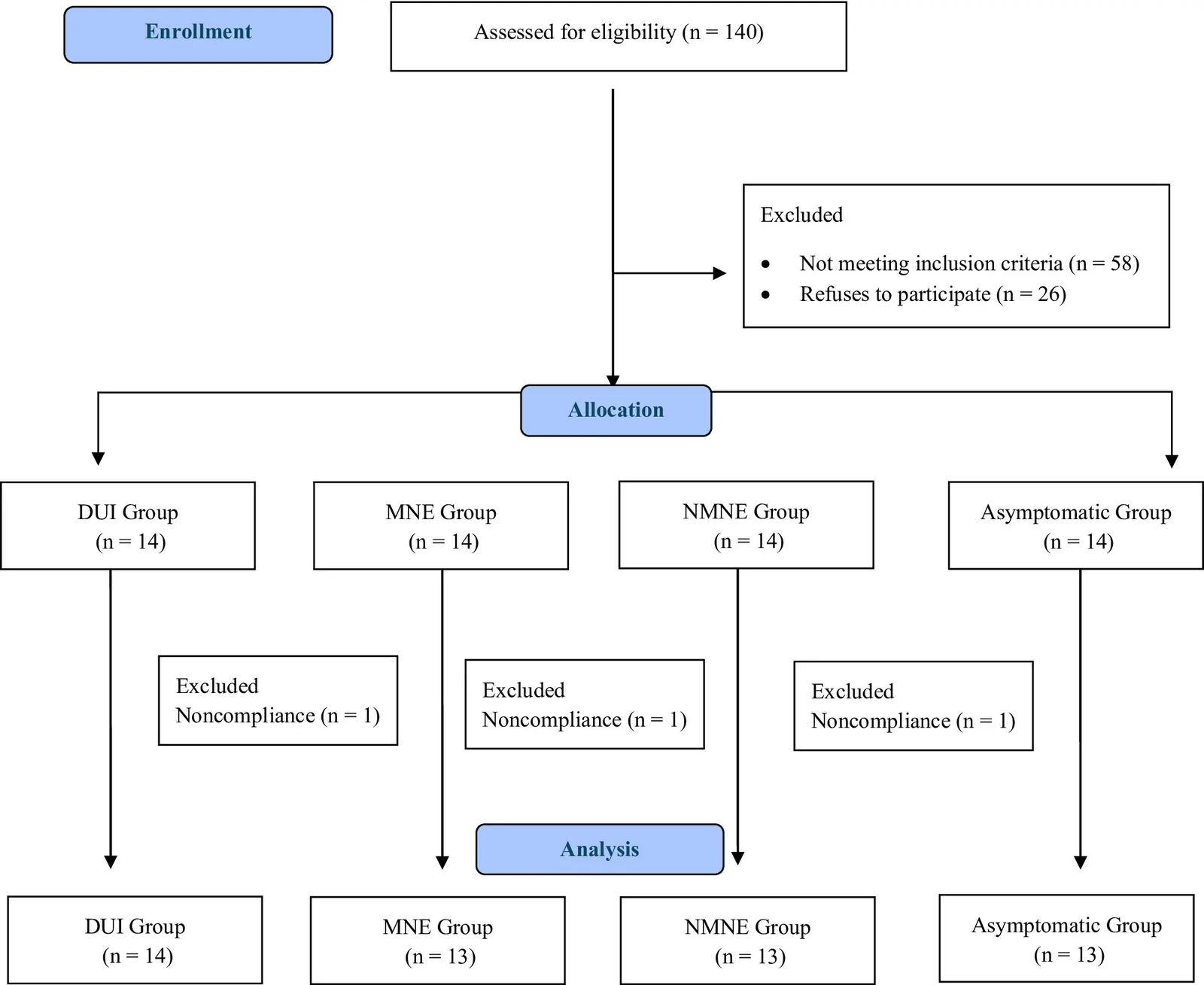
Flow diagram of the study design.

A total of 140 children were screened for eligibility. Of these, 58 did not meet the criteria and 26 declined participation; 56 met the inclusion criteria and were included in the study. The initial distribution of these 56 children among groups was 14 children in each group; however, three children did not complete the full assessment protocol. Consequently, 53 children completed the study and were included in the analysis: 14 in the DUI group, 13 in the MNE group, 13 in the NMNE group, and 13 in the asymptomatic group (see Fig. 1).

No statistically significant demographic differences were found between urinary incontinence subgroups (*p* > 0.05). All three groups had a higher percentage of girls. Groups were also similar in perinatal history, neuromotor development, and habits (*p* > 0.05) (see Table 1).

**Table 1:** Demographic characteristics of children among the urinary incontinence subgroups.

	DUI Group (*n* = 14) Mean ± SD or *n* (%)	MNE Group (*n* = 13) Mean ± SD or *n* (%)	NMNE Group (*n* = 13) Mean ± SD or *n* (%)	P^*^ value
Age (years)	9.38 ± 3.20	8.45 ± 2.01	8.09 ± 2.02	0.666
Sex Female Male	11 (78.57) 3 (21.43)	8 (61.53) 5 (38.47)	9 (69.23) 4 (30.77)	0.262
BMI (kg/m^2^)	19.03 ± 3.62	16.75 ± 3.07	18.14 ± 4.19	0.324
Birth weight (g)	3147.69 ± 636.92	3250.91 ± 392.49	3218.18 ± 541.12	0.863
Gestational age Term Preterm	14 (100.0) 0 (0.00)	13 (100.0) 0 (0.00)	13 (100.0) 0 (0.00)	0.419
Type of delivery Vaginal Cesarean	5 (35.71) 9 (64.29)	4 (30.76) 9 (69.24)	2 (15.38) 11 (84.61)	0.517
Crawling age (months)	7.00 ± 0.57	7.67 ± 2.25	7.60 ± 1.51	0.859
Walking age (months)	12.62 ± 3.25	11.36 ± 1.62	11.18 ± 1.66	0.758
Toilet training age (months)	27.08 ± 7.81	30.00 ± 7.70	23.64 ± 9.11	0.194
Physical activity habit Yes No	6 (42.85) 8 (57.15)	6 (46.15) 7 (53.85)	6 (46.15) 7 (53.85)	0.870
Screen time (min)	195.00 ± 105.09	201.82 ± 97.75	168.00 ± 111.35	0.675
Number of daily meals	4.46 ± 1.05	4.09 ± 1.22	4.09 ± 0.94	0.572
Daily water consumption	1961.54 ± 748.93	1454.55 ± 650.17	2000.00 ± 1204.15	0.148
Consumption of bladder irritants Yes No	11 (78.58) 3 (21.42)	10 (76.93) 3 (23.07)	12 (92.30) 1 (7.70)	0.533
DVISS	14.14 ± 5.33	13.46 ± 5.73	22.26 ± 4.92	**0.001** ^¥^
VBBDQ	15.57 ± 5.62	12.77 ± 4.98	21.31 ± 5.48	**0.001** ^¥^
CBBDQ bladder	16.36 ± 5.09	12.69 ± 7.31	23.31 ± 4.76	**0.001** ^¥^
CBBDQ bowel	5.00 ± 3.38	5.08 ± 3.04	6.85 ± 4.94	0.241
CBBDQ total	21.36 ± 8.90	17.77 ± 8.22	30.15 ± 7.80	**0.003** ^¥^

DUI: Daytime Urinary Incontinence, MNE: Monosymptomatic Nocturnal Enuresis, NMNE: Non-Monosymptomatic Nocturnal Enuresis, BMI: Body Mass Index, SD: Standard Deviation, DVISS: Dysfunctional Voiding and Incontinence Scoring System, VBBDQ: Vancouver Bladder/Bowel Dysfunction Questionnaire, CBBDQ: Childhood Bladder and Bowel Dysfunction Questionnaire.

^*^Kruskal-Wallis test, Chi-square test.

^¥^: *P* < 0.05.

Some postural alignment parameters differed significantly between urinary incontinence subgroups and the asymptomatic group (*p* < 0.05). Left lateral total body angulation was higher in the urinary incontinence groups (*p* = 0.002). Left lateral (*p* = 0.001), right lateral (*p* = 0.045), and posterior (*p* = 0.013) hip-pelvis angulations were also greater, with the highest values in the NMNE subgroup (see Table 2).

**Table 2:** Comparison of the children’s postural alignment parameters among the urinary incontinence subgroups.

Postural Alignment Parameters	DUI Group (*n* = 14) Mean ± SD	MNE Group (*n* = 13) Mean ± SD	NMNE Group (*n* = 13) Mean ± SD	Asymptomatic Group (*n* = 13) Mean ± SD	P^*^ value	Effect size (ε²)	P^**^ value
Left lateral angulation hip-pelvis	4.35 ± 3.00	4.03 ± 3.14	5.16 ± 2.94	0.97 ± 1.47	**0.001** ^¥^	0.316	Asymptomatic vs. MNE = **0.028**^**¥**^ Asymptomatic vs. DUI = **0.008**^**¥**^ Asymptomatic vs. NMNE = **0.001**^**¥**^ MNE vs. DUI = 1.000 MNE vs. NMNE = 1.000 DUI vs. NMNE = 1.000
Left lateral angulation total	17.44 ± 5.76	16.95 ± 7.68	18.13 ± 6.53	9.70 ± 4.21	**0.002** ^¥^	0.284	Asymptomatic vs. MNE = **0.035**^**¥**^ Asymptomatic vs. DUI = **0.008**^**¥**^ Asymptomatic vs. NMNE = **0.005**^**¥**^ MNE vs. DUI = 1.000 MNE vs. NMNE = 1.000 DUI vs. NMNE = 1.000
Posterior angulation hip-pelvis	39.50 ± 75.64	28.97 ± 66.50	42.87 ± 77.16	0.56 ± 0.77	**0.013** ^¥^	0.207	Asymptomatic vs. DUI = 0.090 Asymptomatic vs. MNE = 0.101 Asymptomatic vs. NMNE = **0.014**^**¥**^ DUI vs. MNE = 1.000 DUI vs. NMNE = 1.000 MNE vs. NMNE = 1.000
Right lateral angulation hip-pelvis	4.08 ± 5.10	5.04 ± 2.97	7.04 ± 3.18	3.98 ± 2.55	**0.045** ^¥^	0.155	Asymptomatic vs. DUI = 1.000 DUI vs. MNE = 1.000 DUI vs. NMNE = **0.046**^**¥**^ Asymptomatic vs. MNE = 1.000 Asymptomatic vs. NMNE = 0.196 MNE vs. NMNE = 1.000
Lateral displacements angulation hip-pelvis	3.81 ± 3.61	4.17 ± 2.62	6.10 ± 2.59	2.44 ± 1.61	**0.008** ^¥^	0.227	Asymptomatic vs. DUI = 1.000 Asymptomatic vs. MNE = 0.471 Asymptomatic vs. NMNE = **0.005**^**¥**^ DUI vs. MNE = 1.000 DUI vs. NMNE = 0.121 MNE vs. NMNE = 0.683

DUI: Daytime Urinary Incontinence, MNE: Monosymptomatic Nocturnal Enuresis, NMNE: Non-Monosymptomatic Nocturnal Enuresis, SD: Standard Deviation.

^*^Kruskal-Wallis test.

^**^Dunn’s test with Bonferroni correction.

ε²: Epsilon-squared effect size for the Kruskal-Wallis test, calculated as H/(n-1); ~0.01 small, ~ 0.06 medium, ~ 0.14 large.

^¥^: *P* < 0.05.

In uncorrected analyses, significant correlations were observed between postural alignment parameters and core muscle function in the urinary incontinence subgroups (*p* < 0.05). After Benjamini-Hochberg correction, none of these associations retained statistical significance (q > 0.05). All correlation coefficients, 95% confidence intervals, and corrected q-values are presented in Table 3.

**Table 3:** The relationship between the children’s postural alignment parameters and core muscle functions among the urinary incontinence subgroups.

Group	Core Muscle Functions	Postural Alignment Parameters	*r*	P^*^ value	95% CI	q (BH)
DUI Group (*n* = 14)	PFM pre-baseline rest activity	Left lateral angulation total	−0.604	**0.022**	[−0.864, −0.091]	0.441
	RA rest activity	Left lateral angulation total	−0.563	**0.036**	[−0.847, −0.029]	0.539
	MF rest thickness	Left lateral angulation total	0.659	**0.014**	[0.152, 0.892]	0.324
	IO rest thickness	Left lateral angulation hip-pelvis	0.609	**0.027**	[0.070, 0.873]	0.324
	IO contractile thickness	Left lateral angulation hip-pelvis	0.614	**0.025**	[0.078, 0.875]	0.324
	IO contractile thickness	Posterior angulation hip-pelvis	0.624	**0.023**	[0.093, 0.879]	0.564
	DH displacement	Posterior angulation hip-pelvis	0.596	**0.032**	[0.048, 0.868]	0.564
	TrA contractile activity	Right lateral angulation hip-pelvis	0.727	**0.003**	[0.303, 0.910]	0.097
MNE Group (*n* = 13)	PFM pre-baseline rest activity	Left lateral angulation total	0.653	**0.016**	[0.141, 0.889]	0.186
	PFM rest activity	Left lateral angulation total	0.654	**0.015**	[0.144, 0.890]	0.186
	RA rest thickness	Left lateral angulation total	0.689	**0.009**	[0.206, 0.902]	0.146
	IO rest thickness	Left lateral angulation total	0.761	**0.003**	[0.346, 0.927]	0.090
	DH rest activity	Left lateral angulation total	−0.657	**0.015**	[−0.891, −0.149]	0.186
	DH contractile activity	Left lateral angulation total	−0.560	**0.046**	[−0.854, −0.005]	0.431
	EO rest thickness	Posterior angulation hip-pelvis	−0.702	**0.008**	[−0.907, −0.228]	0.481
	DH rest thickness	Lateral displacements angulation hip-pelvis	−0.606	**0.028**	[−0.872, −0.064]	0.500
	PFM displacement	Lateral displacements angulation hip-pelvis	0.571	**0.042**	[0.010, 0.858]	0.500
	PFM pre-baseline rest activity	Lateral displacements angulation hip-pelvis	−0.565	**0.044**	[−0.856, −0.002]	0.514
NMNE Group (*n* = 13)	PFM displacement	Left lateral angulation hip-pelvis	0.626	**0.022**	[0.097, 0.879]	0.519
	PFM displacement	Posterior angulation hip-pelvis	−0.699	**0.008**	[−0.906, −0.224]	0.498
	EO rest activity	Right lateral angulation hip-pelvis	−0.584	**0.036**	[−0.863, −0.300]	0.435
	MF rest activity	Right lateral angulation hip-pelvis	−0.602	**0.026**	[−0.874, −0.075]	0.435
	PFM displacement	Lateral displacements angulation hip-pelvis	0.684	**0.010**	[0.196, 0.900]	0.476

DUI: Daytime Urinary Incontinence, MNE: Monosymptomatic Nocturnal Enuresis, NMNE: Non-Monosymptomatic Nocturnal Enuresis, RA: Rectus Abdominis, TrA: Transversus Abdominis, IO: Internal Oblique Abdominalis, EO: External Oblique Abdominalis, DH: Diaphragm, MF: Multifidus, SD: Standard Deviation, r: Correlation Coefficient, CI: Confidence Interval.

^*^Spearman’s Rank Correlation test.

*P* < 0.05 (uncorrected).

q (BH)=False discovery rate-corrected *p* value obtained with the Benjamini-Hochberg procedure.

**Fig. 2:**
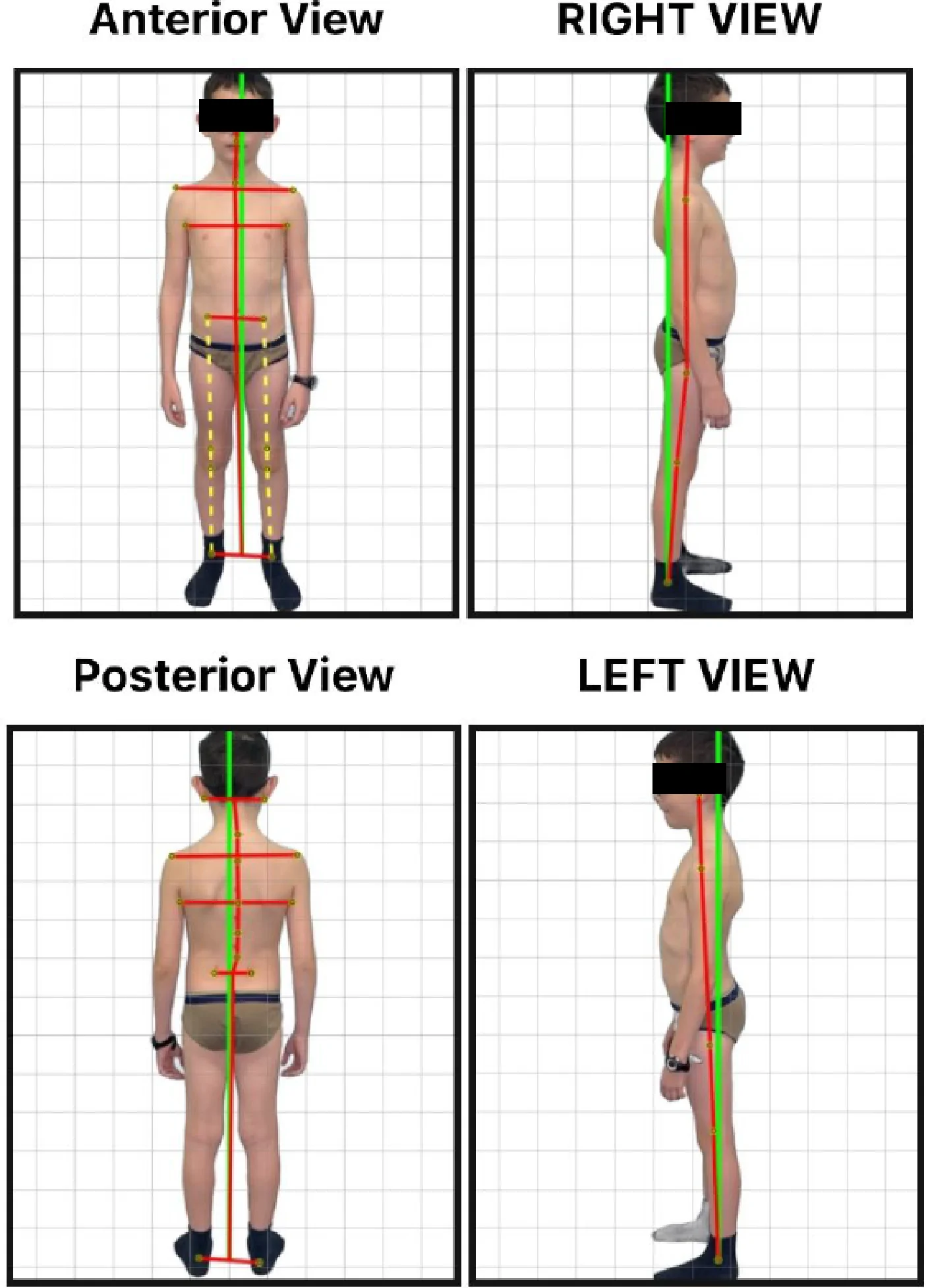
Measurements of the head, shoulder, hip-pelvis, and knee regions from anterior, posterior, and lateral views using the PostureScreen Mobile application (PostureCo, Inc., Trinity, FL, USA), reproduced with permission of the rights holder.

Cluster analysis identified two main clusters (see Fig. 2). Significant differences were found between the lower and greater postural deviation clusters regarding lower urinary tract symptoms and core muscle function (*p* < 0.05). The greater postural deviation cluster had higher CBBDQ bowel (*p* = 0.034) and total (*p* = 0.048) scores, higher resting activity of the pelvic floor muscles (*p* = 0.039), higher transversus abdominis contractile activity (*p* = 0.048), and lower multifidus contractile activity (*p* = 0.013). No significant differences were found in DVISS or VBBDQ total scores between groups. Given the limited sample size and the absence of external validation, these cluster-based results should be regarded as exploratory (see Table 4).

**Table 4:** Comparison of the children’s lower urinary tract symptoms and core muscle functions among the urinary incontinence clusters and the asymptomatic group.

	Cluster 1: Lower Postural Deviation Group (*n* = 26) Mean ± SD	Cluster 2: Greater Postural Deviation Group (*n* = 14) Mean ± SD	Asymptomatic Group (*n* = 13) Mean ± SD	P^*^ value	Effect size (ε²)	P^**^ value
CBBDQ bowel	4.58 ± 4.47	7.57 ± 5.41	-	0.034^¥^	0.336	-
CBBDQ total	20.73 ± 9.49	27.36 ± 8.67	-	**0.048** ^¥^	0.312	-
PFM pre-baseline rest activity	3.18 ± 1.85	3.35 ± 1.89	1.84 ± 0.76	**0.039** ^¥^	0.124	Asymptomatic vs. Cluster 1 = 0.056 Asymptomatic vs. Cluster 2 = 0.053 Cluster 1 vs. Cluster 2 = 1.000
TrA contractile activity	8.59 ± 9.92	14.37 ± 12.92	15.20 ± 15.97	**0.048** ^¥^	0.117	Asymptomatic vs. Cluster 1 = 0.400 Cluster 1 vs. Cluster 2 = 0.066 Asymptomatic vs. Cluster 2 = 1.000
MF contractile activity	4.18 ± 3.27	2.40 ± 1.20	4.70 ± 2.13	**0.013** ^¥^	0.168	Cluster 1 vs. Cluster 2 = **0.046**^**¥**^ Asymptomatic vs. Cluster 2 = **0.026**^**¥**^ Asymptomatic vs. Cluster 1 = 1.000

CBBDQ: Childhood Bladder and Bowel Dysfunction Questionnaire, PFM: Pelvic Floor Muscle, TrA: Transversus Abdominis, MF: Multifidus, SD: Standard Deviation.

^*^Mann-Whitney U test, Kruskal-Wallis test.

^**^Dunn’s test with Bonferroni correction.

ε²: Epsilon-squared effect size for the Kruskal-Wallis test, calculated as H/(n-1); ~0.01 small, ~ 0.06 medium, ~ 0.14 large.

^¥^: *P* < 0.05.

## Discussion

In this study, children with urinary incontinence had significantly greater left lateral total body angulation than asymptomatic children. The lateral and posterior hip-pelvis angulations were also significantly greater. These changes in postural alignment were most pronounced in the children with NMNE. Additionally, significant associations were also found between postural alignment changes and core muscle functions in the urinary incontinence subgroups. Furthermore, the children with greater postural deviations also had more severe lower urinary tract symptoms related to bowel function. In these children, the pre-basal resting activity of the pelvic floor muscles and the contractile activity of the transversus abdominis were higher, while the contractile activity of the multifidus was lower.

Postural alignment changes in the sagittal plane have been reported in children with urinary incontinence. Compared with asymptomatic children, these children have been shown to exhibit greater lumbar lordosis and pelvic anteversion angles^10,11^. In our study, children with urinary incontinence demonstrated greater lateral and posterior hip-pelvis angulations, as well as a greater left lateral total body angulation, than asymptomatic children. These differences were particularly pronounced in the children with NMNE. Our results suggest that postural alignment changes in children with urinary incontinence may extend beyond the sagittal plane to involve the frontal plane, indicating multiplanar postural alignment changes. The more pronounced postural alignment changes observed in the NMNE subgroup may be related to the greater symptom severity in this group. In adults, lumbopelvic postural alignment has been associated with core muscle function, and lumbopelvic stability is known to depend on adequate core muscle strength, endurance, and co-activation^13–20^. In our study, several significant correlations were identified between postural alignment changes measures and core muscle functions among urinary incontinence subgroups. However, none of these associations remained significant after correction for multiple comparisons. Therefore, these results should be interpreted as hypothesis-generating rather than confirmatory. Although our results suggest a potential relationship between postural alignment and core muscle function, the limited subgroup sample sizes warrant cautious interpretation. Larger studies are needed to confirm these results and further investigate the underlying mechanisms.

The observed postural alignment changes, particularly on the left side, cannot be explained in light of our current data. However, neurophysiological findings reported in the literature, while purely speculative, offer a possible explanatory framework for this condition. Central bladder control involves a distributed network including the prefrontal and cingulate cortices, insula, periaqueductal gray matter, and pontine micturition centre, whose dysfunction has been linked to various lower urinary tract dysfunctions^2^. Within this network, the medial prefrontal cortex exerts tonic inhibition that prevents involuntary bladder contractions, and its dysfunction can weaken this control, leading to symptoms such as urgency and urinary incontinence^3,21^. A meta-analysis showed that the right hemisphere is more effective in controlling bladder storage and emptying^22^. These regions involved in bladder control also regulate postural control mechanisms^23^. Similarly, it has been reported that postural control varies with hemispheric localization^24^. Kati et al. reported that the right hemisphere has a dominant role in postural control, and impairments are more severe with right hemisphere lesions^25^. In our study, children with urinary incontinence had greater left lateral total body angulation compared to asymptomatic children. Additionally, our results showed that children predominantly shifted their weight to the right side. It is well established that unilateral hemispheric lesions frequently affect contralateral body segments as well as postural control^26^. Therefore, it is thought that the left lateral postural deviation observed in children with urinary incontinence may be related to right hemisphere networks that play a role in bladder and postural control. However, since neuroimaging was not performed in our study, this hypothesis cannot be tested with our data. Furthermore, the observed asymmetry could also be due to differences in limb dominance or weight-bearing. Therefore, this interpretation is purely hypothetical and should be considered as a research question for future studies.

Studies have shown that pelvic floor muscle activity varies with lumbopelvic posture^13–19^. Lemos et al. found that women with urinary incontinence and a larger anterior pelvic tilt angle had higher pelvic floor muscle activity^18^. Similarly, Martins Reis et al. reported that women with urinary incontinence and myofascial dysfunction had a larger anterior pelvic tilt angle and posterior body displacement^27^. Conversely, Capson et al. found that in continent women, pelvic floor muscle activity was higher during posterior pelvic tilt than during normal or anterior pelvic tilt^14^. Findings in the literature indicate that the relationship between posture and pelvic floor muscle activity is inconsistent. A similar heterogeneity was observed in our study as well. Increased lateral total body angulation appeared to be associated with decreased pelvic floor muscle resting activity in children with DUI and increased activity in those with MNE in uncorrected exploratory analyses. Additionally, greater lateral hip-pelvis angulation was associated with increased pelvic floor displacement in children with MNE and NMNE. These findings suggest that postural asymmetry in the frontal plane can affect both pelvic floor muscle activity and pelvic floor biomechanics, and that these effects vary according to urinary incontinence subtypes. The spine and pelvis are biomechanically interconnected. Spine-pelvis misalignment can create mechanical stress, altering load and pressure distribution in pelvic structures and affecting muscles, fascia, and joints. This may alter pelvic floor muscle activity and potentially contribute to lower urinary tract symptoms like urinary incontinence^18^. The literature suggests that overactive or underactive pelvic floor muscles in children may contribute to bladder and bowel symptoms^28^. Although these correlations were not statistically robust after correction, they may generate hypotheses for future studies examining whether targeting both lateral postural deviation and pelvic floor muscle activity is beneficial. Furthermore, treatment approaches may need to be individualized according to the subtypes of urinary incontinence. In this context, approaches aimed at increasing pelvic floor muscle activity in children with DUI and reducing overactivity in children with MNE could be evaluated in future studies.

The pelvic floor, diaphragm, multifidus, and abdominal muscles work synergistically^29–31^. This synergistic relationship is essential for normal bladder and bowel function^28^. Tuncer et al. reported that children with lower urinary tract dysfunction had lower pelvic floor muscle activity, more severe respiratory muscle weakness, and reduced core muscle endurance compared to asymptomatic children^32^. The literature emphasizes the importance of targeting muscle synergy rather than strengthening each muscle independently^30^. In our study, the correlations differed in direction across subgroups, suggesting that core muscle activation strategies may vary according to incontinence subtype. However, as none of these relationships retained statistical significance after correction for multiple comparisons, this interpretation should be regarded as exploratory.

Bladder and bowel functions are closely linked in children with lower urinary tract dysfunction^33^. In our study, children with urinary incontinence and greater postural deviation had more severe lower urinary tract symptoms related to bowel function. Additionally, these children had higher pre-baseline resting activity of the pelvic floor muscles and greater contractile activity of the transversus abdominis, but lower contractile activity of the multifidus. These results suggest that postural alignment changes may reduce multifidus activation, impair lumbopelvic stability, and increase compensatory activity in the pelvic floor and abdominal muscles. The overactivity of the abdominal muscles in individuals with lower urinary tract dysfunction may prevent pelvic floor muscle relaxation during urination^29,30^. In children, relaxation of the lower abdominal and pelvic floor muscles is important during urination and defecation^31^. Increased abdominal muscle tension has been reported to negatively affect bowel motility in individuals with constipation. However, this relationship is largely based on expert opinion and has not yet been definitively proven^34^. Hence, increased pelvic floor and abdominal muscle activity in children with greater postural deviations might be associated with by affecting bladder and bowel emptying. Physiotherapy programs targeting correct postural alignment, lumbopelvic motor control, and pelvic floor and abdominal muscle relaxation may be beneficial in this population. However, as this is a cross-sectional pilot study, these are hypotheses that require confirmation in prospective interventional trials before they can inform clinical practice.

If our findings are confirmed, posture assessment could be incorporated into the initial urotherapy consultation using the PostureScreen Mobile application, which provides objective measurements of postural deviations. Children with urinary incontinence who exhibit postural deviations may be considered for referral to physiotherapy. However, a validated deviation threshold to guide such referral should be established in future studies. In the current treatment algorithm for pediatric urinary incontinence, standard urotherapy constitutes the first step, and specific urotherapy is applied when an adequate response is not achieved. Rather than replacing these approaches, posture retraining and lumbopelvic motor control training may serve as complementary components alongside them, particularly in children who exhibit postural deviations.

This study is the first to investigate the underlying mechanism of postural alignment changes in children with urinary incontinence at the peripheral level. A major strength is the use of objective, validated methods (PostureScreen Mobile, surface EMG, and ultrasound) to assess postural alignment and core muscle function. However, the PostureScreen Mobile application estimates angles from anatomical reference points and cannot accurately measure spinal curvature. Therefore, direct imaging methods such as spinal X-rays may provide more accurate assessments. As this was a pilot study, the relatively small sample size, particularly in the subgroup analyses (approximately 13 participants per group), reduced the statistical power and may have limited the robustness and generalizability of the results. Therefore, the results are considered exploratory. The cluster analysis in particular was performed without external validation and may be unstable at this sample size. These clusters should therefore be confirmed in larger, independent cohorts before any clinical interpretation. Although the postural alignment changes observed in this study were associated with peripheral core muscle function, possible central mechanisms were not directly evaluated in this study. Further studies involving objective neuroimaging methods and larger sample sizes may help to elucidate central mechanisms.

## Conclusion

This study revealed that children with urinary incontinence have postural alignment differences, particularly in the hip and pelvic regions. These changes were observed to be more pronounced on the left side. In addition, greater postural deviation was also associated with more severe urinary and bowel symptoms. These postural alignment changes appeared to relate to core muscle function at the peripheral level, although this association was based on exploratory correlations that did not remain significant after correction. On the other hand, the predominance of left-sided changes may point to a central contribution to postural and bladder control. However, this remains an untested hypothesis. A multidisciplinary assessment of both peripheral factors (such as postural alignment and core muscle function) and central factors may contribute to the management of these children. Therefore, future neuroimaging studies are needed to examine whether central nervous system mechanisms are involved.

## Methods

### Study design and participants

This cross-sectional study was conducted between October 2025 and January 2026. Forty-two children aged 5–14 years who had been diagnosed with urinary incontinence and met the inclusion criteria were recruited from the Division of Pediatric Urology at the Ege University Faculty of Medicine Hospital’s Department of Pediatric Surgery. Fourteen asymptomatic children without lower urinary tract symptoms were also recruited from the Department of Pediatrics at the same hospital.

The inclusion criteria for the urinary incontinence group were age 5–14 years, diagnosis by a pediatric urologist according to ICCS criteria, signed informed consent, and sufficient Turkish proficiency (by parent or child) to complete questionnaires. Controls were aged 5–14 years, provided informed consent, and had no history of lower urinary tract symptoms.

The exclusion criteria for both groups were neurogenic lower urinary tract dysfunction, anatomical urinary tract abnormalities, active urinary tract infection, prior surgical intervention for incontinence, conditions preventing participation (e.g., cognitive or orthopedic impairment), chronic diseases (e.g., chronic kidney disease, diabetes, hypertension, cancer), congenital thoracic deformities, and participation in exercise therapy within the past six months.

## Ethics statement

**Fig. 3:**
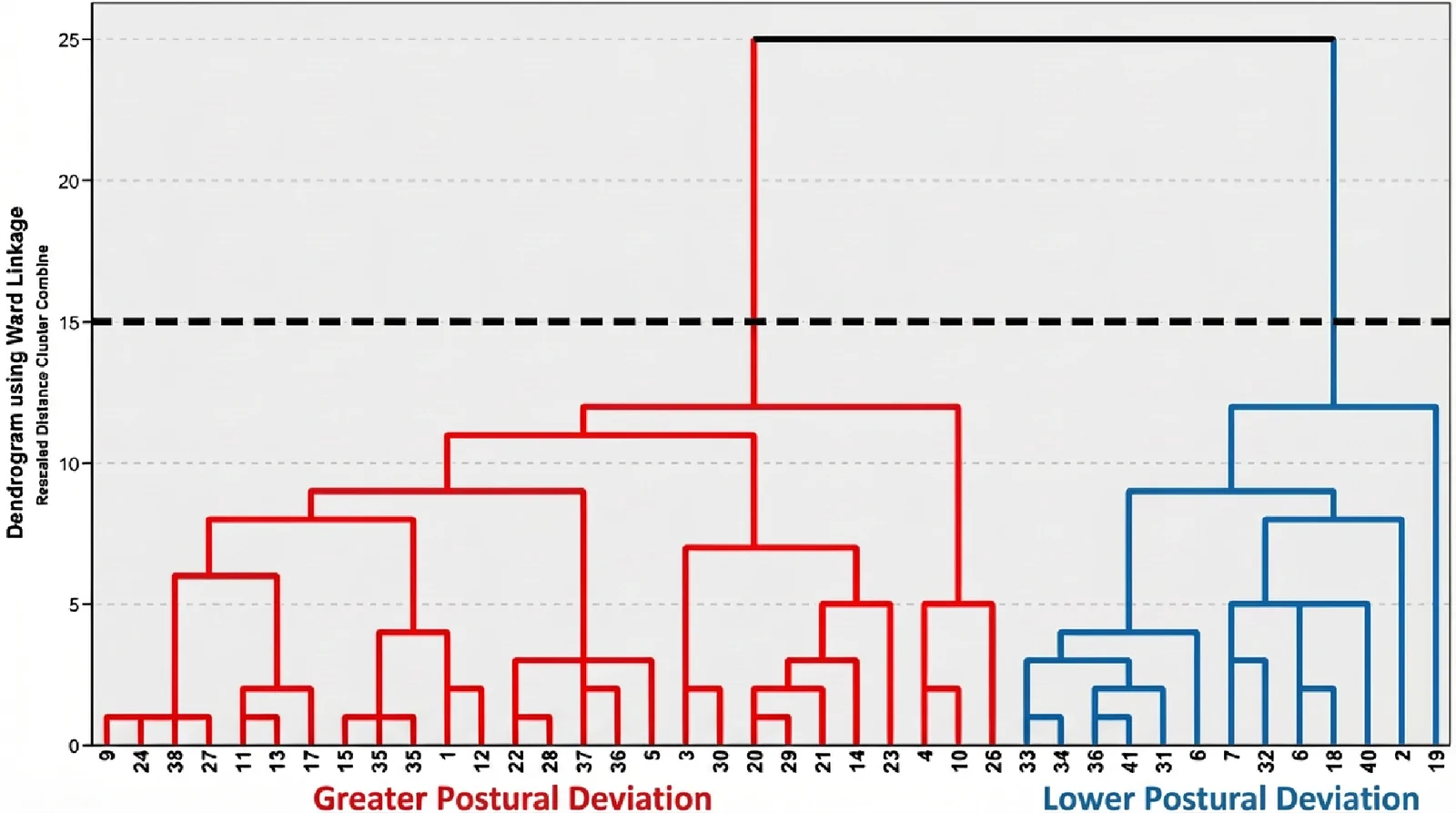
Dendrogram of the cluster analysis (different colors show the different clusters).

The study was conducted in accordance with the ethical standards set out in the Declaration of Helsinki and was approved by Dokuz Eylul University’s Institutional Non-invasive Research Ethics Committee (Approval No: 2025/04–09, File No: 9531-GOA). All participants and their parents provided written informed consent after receiving verbal and written information. Written assent was also obtained from children aged 7 years and older. Written informed consent for the publication of identifying images in a publication was obtained from the parent of the child whose images appear in Fig3.

### Sample size and power calculation

A priori sample size calculation was not feasible due to the lack of available prior data. As this was a pilot study, effect sizes (ε²) are reported alongside each comparison in the tables and may be used to inform sample-size planning for future adequately powered studies. The subgroup, correlation, and cluster analyses remain underpowered and are therefore presented as exploratory.

## Blinding and reliability

Assessments were divided between two researchers specialized in pelvic floor rehabilitation, both blinded to group allocation. One researcher performed the symptom questionnaires and postural alignment assessment, while the other performed the surface EMG and ultrasound assessments. As each assessment domain was performed by a single dedicated rater, intra- and inter-rater reliability was not evaluated. As the postural measurements involved manual landmark identification, they are subject to measurement error that we cannot quantify. This may particularly affect the smaller between-group differences, whereas the more pronounced hip-pelvis differences are less likely to be explained by measurement error alone. Future studies should establish rater reliability and use repeated measurements.

## Assessments

Urinary incontinence was diagnosed by a pediatric urologist according to ICCS criteria. Children were classified into DUI, MNE, and NMNE subgroups^1^. The children’s sociodemographic characteristics (age, sex, body mass index, lifestyle habits, gestational age, birth weight and type, motor milestones, and toilet training age) were recorded. The postural alignment of the children was assessed using the PostureScreen Mobile application. The evaluation of lower urinary tract symptoms was conducted using the Dysfunctional Voiding and Incontinence Scoring System (DVISS), the Vancouver Bladder/Bowel Dysfunction Questionnaire (VBBDQ), and the Childhood Bladder and Bowel Dysfunction Questionnaire (CBBDQ). Core muscle function was measured by surface electromyography and ultrasound.

### Assessment of postural alignment

Children’s postural alignment was assessed using PostureScreen Mobile (PostureCo, Inc., Trinity, FL, USA) on an Apple iPhone 15. Data were collected between October 2025 and January 2026. During this period, the application was updated routinely and versions 14.3 to  15.4 were used. Anatomical landmarks were digitised manually by the same assessor for all participants. The PostureScreen Mobile^®^ application is a portable, noninvasive, and validated tool for evaluating static posture^35,36^. The application was used under a valid paid subscription licence held by the research team. Assessments were conducted in a standardized room, with children standing on marked tape while photographs were taken from anterior, posterior, and lateral views. The smartphone was placed 3 m from the tape and 1.5 m high. Children adopted a comfortable posture with their gaze forward. Images were annotated using anatomical reference points, and postural deviations of the head, shoulders, trunk, hip-pelvis, and knees were calculated in degrees from these views. Ideal alignment was defined as zero deviation. This study reports measurements for the head, shoulders, hip-pelvis, and knees from all perspectives (see Fig2).

### Assessment of lower urinary tract symptoms

The DVISS is a questionnaire that assesses the presence and severity of lower urinary tract symptoms in children aged 4–10, completed by their parents. It consists of 14 questions covering daytime and nighttime symptoms, bladder and bowel habits, and quality of life. Scores range from 0 to 35, with higher scores indicating more severe symptoms, and the cut-off point is 8.5. The DVISS has 90% sensitivity and specificity for detecting lower urinary tract dysfunction; however, it does not assess the frequency or severity of constipation. The scale’s Cronbach’s alpha is 0.72^37,38^.

The VBBDQ consists of 13 specific questions and 1 general question, rated on a 5-point Likert scale (0–4). It covers symptoms such as incontinence, voiding, enuresis, dysuria, and constipation. The general question is not included in the total score, which ranges from 0 to 52. Higher scores indicate greater symptom severity, and a score of 11 or above is indicative of bladder and bowel dysfunction. Cronbach’s alpha is 0.727, reflecting acceptable internal consistency^39–41^.

The CBBDQ is a questionnaire that assesses bladder and bowel dysfunction in children aged 5–12. It contains 18 items, which are divided into a 10-item Bladder Symptoms Scale and an 8-item Bowel Symptoms Scale. Parents rate symptoms experienced over the past month on a 5-point Likert scale (0–4). Total scores range from 0 to 72; higher scores indicate more severe symptoms. A cut-off score of 11 yields 96% sensitivity and 89% specificity for identifying dysfunction. Cronbach’s alpha values are 0.74 for the bladder subscale and 0.71 for the bowel subscale^42–44^.

### Assessment of core muscle function

Surface electromyography (EMG) was used to assess core muscle function by recording the bioelectrical activity of muscle fibres, which is an indicator of neuromuscular function^45^. Surface EMG is a valid and reliable method for evaluating the pelvic floor and other core muscles^46,47^. In this study, bioelectrical activity was measured using a NeuroTrac MyoPlus 4 PRO EMG device (Verity Medical Ltd., UK). Technical details are provided in our previous publication^48^.

The bioelectrical activity of the core muscles was assessed using disposable, self-adhesive Ag/AgCl surface electrodes with a diameter of 3.2 cm. The skin was cleaned with alcohol to reduce impedance^49^. To maintain contact and prevent artefacts, a hypoallergenic, high-adhesion medical tape was applied over the electrodes. Three surface electrodes were used for each muscle. The reference electrode was placed at the anterior superior iliac spine (ASIS)^50^. The active electrodes were positioned on the right side of the body, in line with the muscle fibers and in accordance with the reference points specified in the literature^51–53^:

- Pelvic floor muscles: At the 3 o’clock and 9 o’clock positions over the perianal region,
- Rectus abdominis: 3 cm above the umbilicus and 3 cm lateral to the midline,
- Transversus abdominis: 2 cm above the midpoint of the line between the pubic symphysis and the ASIS.
- Internal oblique abdominals: Approximately 2 cm above the inguinal ligament on the mid-clavicular line, at the midpoint between the ASIS and the pubic symphysis.
- External oblique abdominals: At the midpoint of the lowest subcostal angle on the mid-axillary line.
- Diaphragm: 2 cm lateral to the umbilicus, 7th and 8th intercostal spaces from the anterior axillary line.
- Multifidus: Lateral to the spinous process at the L5-S1 level.

Before the measurement, the children were asked to empty their bladders completely.

The children were taught about the anatomy and function of the pelvic floor muscles and were shown how to contract and relax them properly using external palpation and biofeedback. During the assessment, the children lay on their backs with their knees flexed at 140°, their feet on the bed, and their thighs/feet about 30 cm apart. They were instructed to relax or contract their pelvic floor muscles on command, while avoiding activating their abdominal, hip, or thigh muscles, drawing in their abdomen, or holding their breath^54^.

The pelvic floor muscle assessment included pre-baseline resting activity, tonic activity, endurance duration and the number of phasic contractions. Only tonic activity was evaluated for the other core muscles. Resting activity was measured over a period of 30 s. Tonic activity was assessed using three maximal voluntary contractions, each lasting five seconds and followed by five seconds of relaxation. The device recorded the average value in microvolts (µV) during contractions and relaxation. Surface EMG measurements were performed in accordance with the 2021 International Continence Society report on the assessment of pelvic floor muscle function^45^, and the terminology used by Pekbay et al^54^..

Transabdominal ultrasound is a valid, reliable, and noninvasive method for assessing pelvic floor muscle function in children with lower urinary tract dysfunction^1,55^. The SonoScape SSI 600 ultrasound machine was used to assess pelvic floor and diaphragmatic displacement, as well as the thickness of the rectus abdominis, transversus abdominis, internal and external oblique abdominal muscles, multifidus, and diaphragm. To assess pelvic floor displacement, the child was positioned supine. Measurements were taken when the child indicated the urge to urinate and while the bladder was full. The probe was positioned at a 15–30 degree angle over the suprapubic region to visualize the pelvic floor, using the bladder base as an indicator of pelvic floor movement. The degree of pelvic floor displacement was calculated from freeze-frame ultrasound images. Initially, a marker was positioned on the bladder base in a state of repose. Subsequently, the child was requested to voluntarily contract their pelvic floor muscles for 5 s. At this point, the image was frozen in order to place a second marker. The distance between the two markers was measured in millimeters. During voluntary contraction, pelvic floor movement was recorded as cranial or caudal^1,56^.

The thicknesses of the transversus abdominis and internal/external oblique muscles were measured by placing the ultrasound probe perpendicular to the anterolateral abdominal wall between the iliac crest and 12th rib^57^. The rectus abdominis thickness was assessed 2 cm lateral to the umbilicus^58^. Multifidus muscle thickness was measured by positioning the probe longitudinally at L4, then laterally to the L4-L5 facet joint^59^. Measurements were taken at end-expiration and during a five-second pelvic floor contraction, from the lower to the upper fascial lines. To measure diaphragm thickness, the probe was fixed to the chest wall and B-mode imaging was used. Then, M-mode was used to take measurements at end-expiration while the child breathed quietly. All parameters were measured three times and the mean was calculated and recorded^60,61^.

### Statistical analysis

The statistical analysis was conducted using IBM SPSS Statistics 21.0 (IBM Corp., Armonk, NY, USA). The Kolmogorov-Smirnov/Shapiro-Wilk test was used to assess the normality of the data distribution. Continuous variables were presented as mean ± standard deviation, and categorical variables as frequency and percentage [n (%)]. Differences in postural alignment parameters between urinary incontinence subgroups and the asymptomatic group were analyzed using the Kruskal-Wallis test. Spearman’s correlation analysis was used to assess the relationship between postural alignment parameters and core muscle function in each urinary incontinence subgroup. Because a large number of correlations were computed, the p-values were corrected for multiple comparisons using the Benjamini-Hochberg false discovery rate (FDR) method. The correction was applied separately within each subgroup, with all correlations computed in a given subgroup treated as a single family of tests (*n* = 60 per subgroup). Given the limited subgroup sample sizes, the correlation findings were interpreted as exploratory and hypothesis-generating observations rather than confirmatory results.

Cluster analysis was used to identify subgroups of postural alignment, employing both hierarchical (Ward’s method) and non-hierarchical (K-means) clustering methods. Initially, deviation degrees of the head, shoulders, and hip-pelvis from anterior, lateral, and posterior views were converted into Z-scores. The appropriate number of clusters was then determined by examining the dendrogram obtained through hierarchical clustering. The final clusters were labeled ‘lower postural deviation’ and ‘greater postural deviation’ according to the mean variable values, with observations assigned using the K-means method. Participants with low Z-scores were categorized into the lower postural deviation group. This group was considered to have more ideal postural alignment compared to the overall sample. Participants with high Z-scores were included in the greater postural deviation group. This group exhibited more pronounced postural deviations in the head, shoulder, and hip-pelvis regions. Differences between clusters in lower urinary tract symptoms and core muscle function were analyzed using the Mann-Whitney U test. A p-value of < 0.05 was considered statistically significant in all analyses.

## Data Availability

The data is available upon reasonable request from the corresponding author.

## Code availability

No custom code, algorithm, or software pipeline was generated or used in this study. All analyses were performed using commercially available standard software IBM SPSS Statistics 21.0 (IBM Corp., Armonk, NY, USA) and postural measurements were obtained using the commercial application PostureScreen Mobile (PostureCo, Inc., Trinity, FL, USA). No new software, tool, or algorithm was developed.

## Declarations

### Third-party materials and permissions

The DVISS, VBBDQ and CBBDQ were administered in their previously validated Turkish versions without any modification to items, wording, response options or scoring. Written permission for their use was obtained from the instrument developers and from the authors of the Turkish validation studies. No questionnaire items, scoring instructions, forms or substantial excerpts are reproduced in this manuscript or in any supplementary material. PostureScreen Mobile^®^ was used under a valid paid subscription licence permitting research use. Written permission to reproduce the application-generated images in Fig. 2, including under the CC BY 4.0 licence, was obtained from PostureCo, Inc.
